# Forecasting Temporal Dynamics of Cutaneous Leishmaniasis in Northeast Brazil

**DOI:** 10.1371/journal.pntd.0003283

**Published:** 2014-10-30

**Authors:** Joseph A. Lewnard, Lara Jirmanus, Nivison Nery Júnior, Paulo R. Machado, Marshall J. Glesby, Albert I. Ko, Edgar M. Carvalho, Albert Schriefer, Daniel M. Weinberger

**Affiliations:** 1 Department of Epidemiology of Microbial Diseases, Yale School of Public Health, New Haven, Connecticut, United States of America; 2 Serviço de Imunologia, Hospital Universitário Prof. Edgard Santos, Universidade Federal da Bahía, Salvador, Brazil; 3 Center for Women's Health and Gender Biology, Brigham and Women's Hospital, Boston, Massachusetts, United States of America; 4 Centro de Pesquisas Gonçalo Moniz, Fundação Oswaldo Cruz, Ministério da Saúde, Salvador, Brazil; 5 Division of Infectious Diseases, Weill Cornell Medical College, New York, New York, United States of America; Universidad de Buenos Aires, Argentina

## Abstract

**Introduction:**

Cutaneous leishmaniasis (CL) is a vector-borne disease of increasing importance in northeastern Brazil. It is known that sandflies, which spread the causative parasites, have weather-dependent population dynamics. Routinely-gathered weather data may be useful for anticipating disease risk and planning interventions.

**Methodology/Principal Findings:**

We fit time series models using meteorological covariates to predict CL cases in a rural region of Bahía, Brazil from 1994 to 2004. We used the models to forecast CL cases for the period 2005 to 2008. Models accounting for meteorological predictors reduced mean squared error in one, two, and three month-ahead forecasts by up to 16% relative to forecasts from a null model accounting only for temporal autocorrelation.

**Significance:**

These outcomes suggest CL risk in northeastern Brazil might be partially dependent on weather. Responses to forecasted CL epidemics may include bolstering clinical capacity and disease surveillance in at-risk areas. Ecological mechanisms by which weather influences CL risk merit future research attention as public health intervention targets.

## Author Summary

Cutaneous leishmaniasis (CL) is a disease resulting from infection by the *Leishmania* parasites, which humans may acquire when bitten by an infected sandfly. From a public health standpoint, it is important to identify cases early and monitor patients' clinical outcomes because unsuccessfully-treated patients are at risk for severe complications. Since weather conditions affect survival and reproduction of sandflies that transmit *Leishmania*, routinely-gathered weather and climate data may be useful for anticipating CL outbreaks, bolstering clinical capacity for high-risk periods, and initiating interventions such as active case-finding during these periods to limit disease burden. Here we assessed whether the number of CL cases occurring per month in a rural region of Bahía, Brazil was associated temperature, humidity, precipitation, and El Niño sea surface temperature oscillation patterns observed during preceding seasons. We formulated models that improved accuracy of one, two, and three month-ahead CL predictions by accounting for weather. Forecasts of this nature can contribute to reducing CL burden by informing resource allocation and intervention planning in preparation for epidemics.

## Introduction

Diseases caused by the *Leishmania* parasites, including cutaneous leishmaniasis (CL), are important in tropical and subtropical areas worldwide, causing over one million cases per year [1]. Although the burden of leishmaniasis in the Americas has reportedly decreased [2], areas of northeastern Brazil, where *Leishmania (Viannia) braziliensis* is endemic, have seen increasing CL case notifications in recent decades [3], [4]. Recurring epidemics in this region comprise an increasing component of overall CL burden in Brazil [5], [6]. The endemic area is additionally expanding eastward from its historical center in the interior *cerrado* uplands toward coastal Atlantic forests [7], [8].

The increase in CL incidence and geographic range expansion by *L. (V.) braziliensis* are significant public health concerns. While CL does not cause death in the absence of complications, the disease causes debilitating and stigmatizing lesions and may progress to dangerous manifestations including mucosal or disseminated infection if treatment is not initiated early in the clinical course [9]–[11]. Individuals infected with *L. (V.) braziliensis* are more likely than other CL victims to experience such complications, which have been observed with increasing frequency in northeastern Brazil over the last three decades [3], [7], [9]. These trends are problematic because current chemotherapeutic regimens for CL have limited efficacy and because an increasing proportion of *L. (V). braziliensis* infections are resistant to first-line antimonial treatment [7], [12]–[14].

Forecasting CL epidemics could aid the allocation of public health resources in advance of high-risk periods [15]. Poor understanding of *L. (V.) braziliensis* has historically hindered efforts to anticipate CL risk in Brazil [16]–[18]. However, as for other vector-borne infections, variations in rainfall and temperature might be associated with outbreaks [15], [19]–[22]. Seasonal and weather-dependent population dynamics of insect vectors that transmit CL in South America motivate consideration of climatic and meteorological factors that may drive disease incidence [23]–[29]. Recent studies have demonstrated that local meteorological observations and global climate patterns such as the El Niño Southern Oscillation improve CL forecasting in Costa Rica [15], [19], [22], [30]. Although correlations between weather and CL [31] or visceral leishmaniasis (VL) [32] have been documented elsewhere, these observations have not yet led to the development disease forecasting systems serving most populations at risk [15]. In this study, we sought to identify potential associations between weather and CL risk and used these findings to develop model-based early warning systems for CL in a region of Northeast Brazil with endemic *L. (V.) braziliensis* transmission.

## Materials and Methods

### Disease data

The Corte de Pedra health post in Presidente Tancredo Neves, Bahía, Brazil maintained paper records for leishmaniasis cases treated from 1988 onward. The health post treats over 90% of CL cases from surrounding municipalities; although the area has historically supported *L. amazonensis*, only *L. (V.) braziliensis* has been isolated from CL patients in the past two decades [7], [8], [14], [33], [34]. We used an aggregated time series comprising 10% of leishmaniasis cases identified at the health post; the dataset, and epidemiologic and clinical summaries of the cases, are described in a previous article [7]. Institutional review boards of the Federal University of Bahia and Weill Cornell Medical College approved the human subject protocol for the original study. We considered only CL cases to avoid double-counting CL patients progressing to disseminated or mucosal infection after initial treatment and to minimize heterogeneity in latent and pre-treatment periods.

### Meteorological data

We obtained daily ground-surface meteorological observations from all weather stations within a 500 km radius of the health post, as reported through the historical databank of the Instituto Nacional de Meterologia (INMET; http://www.inmet.gov.br/portal/). Daily meteorological data were available from 11 weather stations in and adjacent to Bahía as listed in the supplement (**Table S1**). Data from the weather stations were sparse prior to 1992. To allow consideration of lags up to 24 months in length between weather exposures and disease outcomes, we considered only cases presenting for treatment from 1994 onward. To monitor ENSO variations, we used the monthly Multivariate ENSO Index (MEI) [35], which quantifies meteorological anomalies related to variations in sea surface temperature in Niño Region 3.4 of the Pacific Ocean (5°N–5°S, 120°–170°W). Since MEI is computed as a two-month running average, we matched the disease cases in the current month with the MEI that covered the current and previous month.

Because the location of the weather stations does not necessarily match the study area, we interpolated [36] the time series of meteorological data for the study location based on the surrounding weather stations. We describe the interpolation procedure in detail in the supplemental methods (**Text S1**). Using these interpolated time series, we calculated the expected mean noontime temperature (°C), relative humidity (%), days with rainfall (%), and total daily rainfall (cm) within each municipality in the study area for each month over the period 1992–2008. To aggregate values at the regional level, we took the mean interpolated value for each month across all municipalities.

### Time series modeling

**Figure 1:**
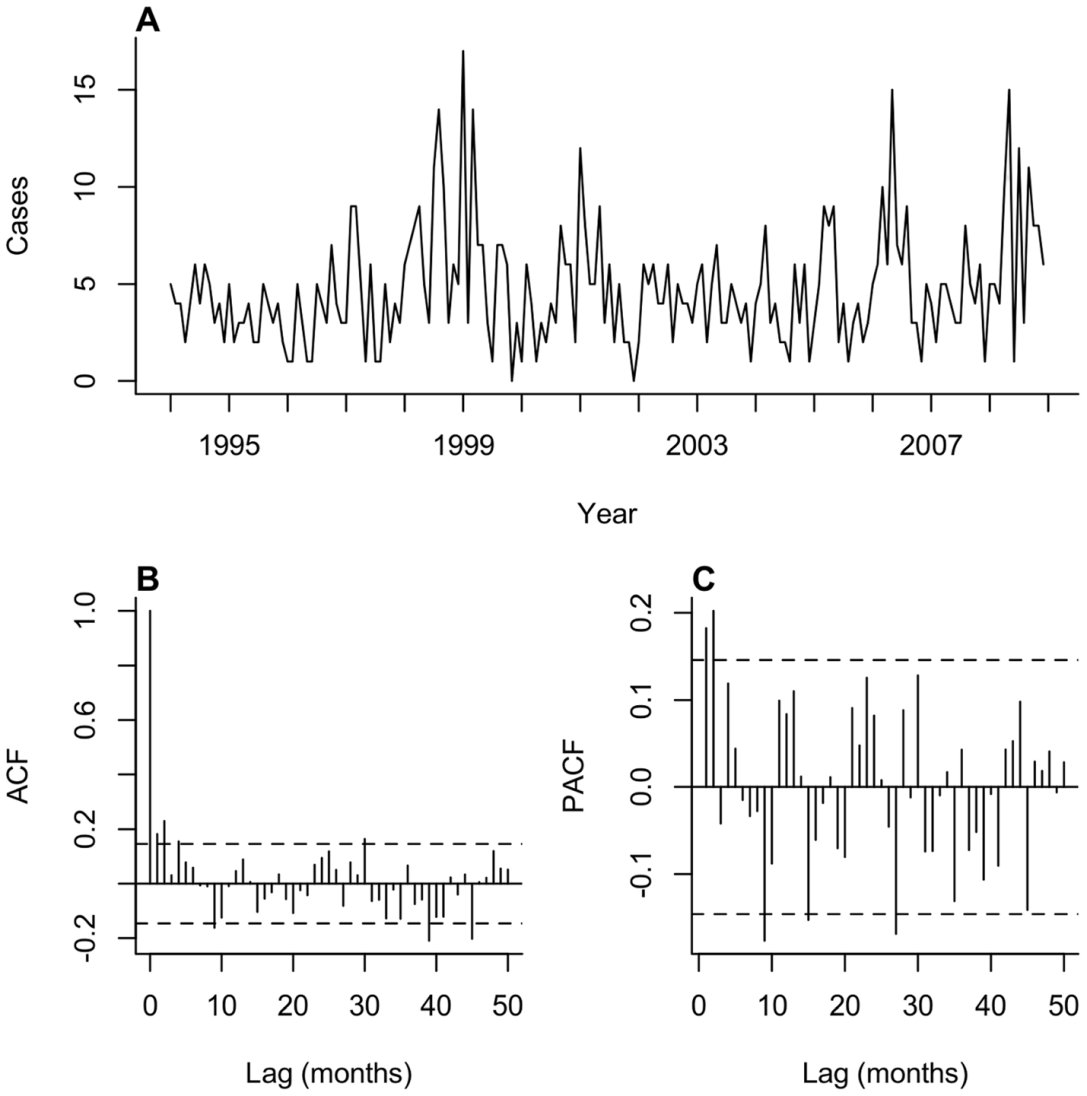
Cutaneous leishmaniasis cases in the study region, 1994–2008. (A) Cases presenting to the Corte de Pedra health post, aggregated by month; (B) Autocorrelation function computed from the square root-transformed case series during the training period; (C) Partial autocorrelation function computed from the square root-transformed case series during the training period. For (B) and (C): the dotted line indicates the 95% significance cut-off.

**Figure 2:**
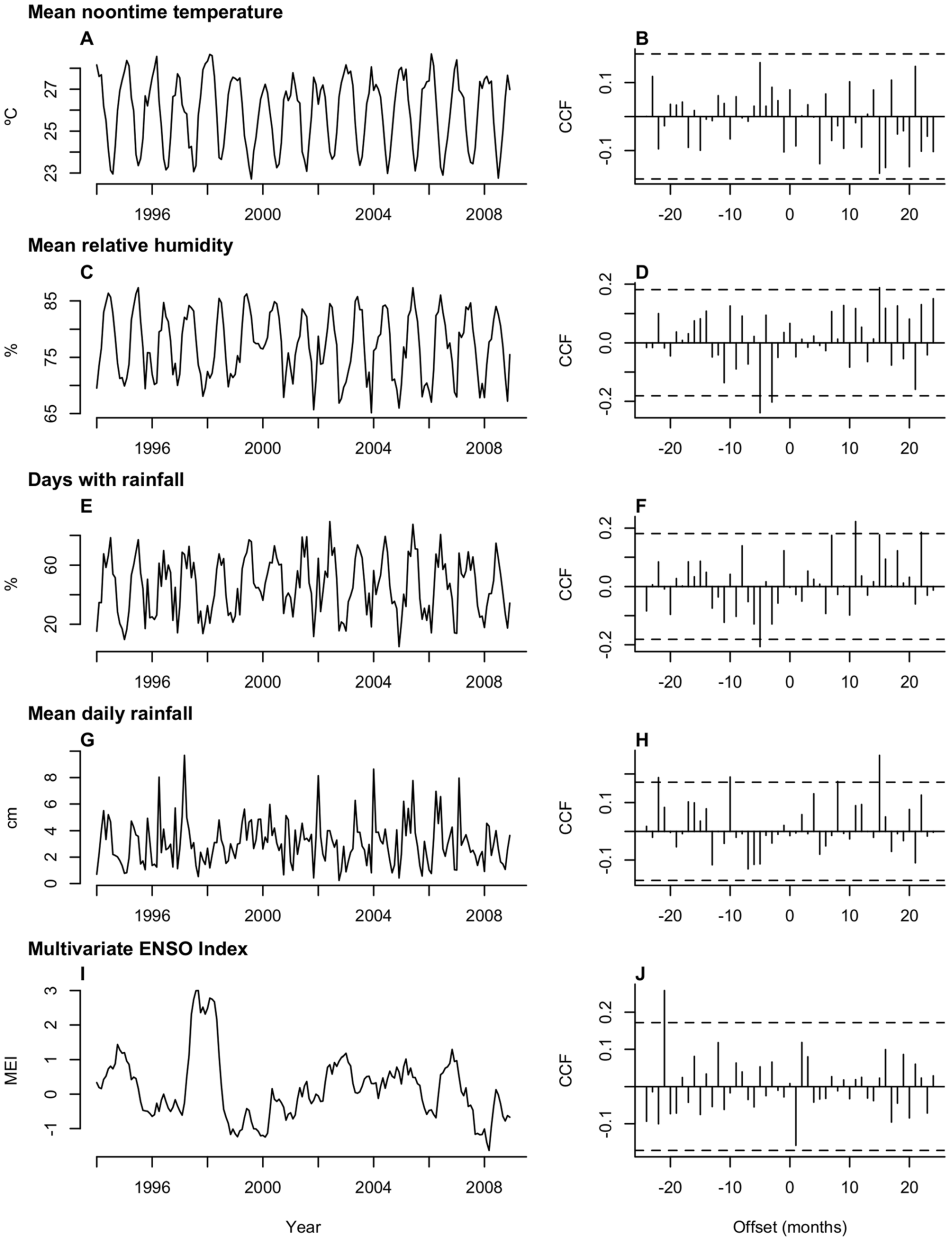
Meteorological and climatic predictors, 1994–2008. Panels for each variable include (right) the interpolated time series for meteorological and climate conditions in the study region, and (left) the cross-correlation with the square root-transformed case series during the training period, in which the dotted line indicates the 95% significance cut-off. The X-axis gives the time separating the meteorological observation from the month of case notification; negative X values indicate lags (weather precedes cases), while positive values indicate leads.

We normalized the time series of monthly CL cases by taking the square root. We identified an autoregressive integrated moving average (ARIMA) or seasonal ARIMA (SARIMA) specification for a null model describing temporal dependence in the transformed case series. Formal descriptions of the ARIMA and SARIMA frameworks, and procedures for model identification, are presented elsewhere [37], [38]. We determined an appropriate order for non-seasonal and seasonal autoregressive, integrated, and moving-average parameters in the null model according to three factors: (1) we identified significant lags in the autocorrelation and partial autocorrelation functions computed from the time series (**Figure 1**); (2) we ensured residuals from the null models did not retain significant temporal autocorrelation based on the Ljung-Box test [39] and inspection of the autocorrelation and partial autocorrelation functions computed from the residuals; and (3) we investigated potential overfitting relative to simpler order specifications according to the Akaike and Bayesian Information Criteria (AIC and BIC) [40], [41].

We used a common pre-whitening approach to select lags of the predictors to be used as covariates in forecasting models [37], [42]. The first step involved fitting a unique (S)ARIMA model to each predictor variable (*X_i_*) on the basis of the variable's autocorrelation and partial autocorrelation functions, reducing the residuals of the *X_i_* input to white noise. We used the fitted models for the predictors to filter the transformed case series (*Y*). We computed the cross-correlation function (CCF) between the residuals of the *Y* and *X_i_* series and tested for significance at the 95% confidence level (cut-off at 1.96*n* ^−1/2^, where *n* was the length of the time series in months). We considered as covariates all lags of the *X_i_* variables where the absolute value of the CCF between the filtered series exceeded the cut-off.

We partitioned the data into an initial “training” period comprising observations for the interval ending in 2004 (132 months), and a “validation” period for the remaining 48 months from 2005 to 2008. The data from the training period served as a basis for estimating the initial autocorrelation and partial autocorrelations to be used for time-series modeling and lag filtering. We parameterized models to fit the training data and used the fitted models to forecast the number of cases in future time periods. The model fit was updated iteratively with the next most recent month, and new forecasts were generated based on the updated models. We generated forecasts at predictive horizons ranging from one month to the maximum number of months ahead that would be possible to predict from incoming data; the shortest significant lag in the CCFs thus specified the maximum forecast horizon (3 months). We centered and scaled all covariates prior to modeling by subtracting their means and dividing by their standard deviations; this allows parameters to be interpreted in terms of covariate standard deviation units to facilitate comparison of effect sizes [43], [44]. As a linear transformation of the covariates, this maintains a linear functional form relating measured predictors to square root-transformed cases. Models predicting square root-transformed CL cases using linear and non-linear relations to meteorological covariates have been compared in previous studies [15]. We ensured via the Ljung-Box test, and by checking autocorrelation and partial autocorrelation functions computed from model residuals, that introducing covariates did not induce temporal dependence in model residuals.

### Multi-model inference

We considered several potential forecasting models for CL. First, we generated a null (S)ARIMA model predicting the transformed case series on the basis of its temporal dependence patterns alone. We additionally generated regression models considering all possible combinations of covariates, and fit each model with the null (S)ARIMA error specification determined from the ACF and PACF of the transformed case series. Last, we used Bayesian model averaging [41] to pool parameter estimates from the fitted models and formulate a global model. We calculated model weights (posterior probabilities for each fitted model) via the AIC, AICc, and BIC and used the weights to pool parameter, variance, and covariance estimates, as described elsewhere [41]. In addition to providing parameter estimates, the model averaging approach can be used to calculate the posterior probability that each covariate is useful in predicting monthly CL cases; this value is given as the sum of posterior model probabilities for models that included the covariate (we refer to parameter posterior probability as PPP henceforward). For model averaging, we updated posterior weights at each time point as models were re-fitted to incoming data. We conducted sensitivity analyses without updating of weights to verify certainty in the results.

We evaluated models' predictive accuracy on the basis of MSE in predictions; we computed this value by comparing model forecasts to the square root-transformed cases observed during the validation period. We compared predictive accuracy for models with covariates relative to the null model to ascertain improvements in forecasting.

## Results

### Epidemiologic characteristics

The dataset included 1,209 leishmaniasis cases treated at the Corte de Pedra health post between 1988 and 2008. We identified 853 cases without disseminated or mucosal infection presenting for care between 1994 and 2008. Of these, 586 occurred in the initial training period (1994–2004) and 267 occurred in the validation period (2005–2008). The most notable epidemic appeared in 1999–2000 (**Figure 1**). The majority of cases occurred among adult male agricultural workers. The median age at symptom onset was 22, and the age distribution was heavily skewed toward younger ages. Further epidemiologic and clinical details about the cases are available elsewhere [7].

The transformed case series had a stationary mean indicating differencing was not required. The autocorrelation function showed significant dependence extending to a four-month lag, while significant partial autocorrelation cut off after a two-month lag (**Figure 1**). We identified no evidence for recurring seasonal patterns in the autocorrelation and partial autocorrelation functions. AIC and BIC scores indicated that accounting for autoregressive or moving average dependence at four-month lags resulted in model overfitting, as did incorporating a 12-month autoregressive term in a SARIMA framework. According to these observations and on the basis of eliminating autocorrelation in the residuals as detected by the Ljung-Box test and residual series' autocorrelation and partial autocorrelation functions, we selected an ARIMA(2,0,3) framework for the null model.

### Meteorological predictors

We identified significant cross-correlations between the case series and all predictors except temperature (**Figure 2**). The three-month lag at which relative humidity and CL cases were significantly correlated provided the maximum forecast horizon. We identified significant, negative-valued cross correlations linking pre-whitened CL cases to relative humidity and rainfall frequency at lags between three and five months (**Table 1**). We identified significant, positive cross-correlations with MEI (22-month lag) and total rainfall (10- and 21-month lags, respectively).

**Table 1:** Covariate lag selections and model parameter estimates.

			Null model	Best-fit (BIC)	Best-fit (AIC/AICc)	Averaged (BIC)		Averaged (AIC/AICc)	
	Lag	CCF	Est. [95% CI]	Est. [95% CI]	Est. [95% CI]	Est. [95% CI]	PPP (%)	Est. [95% CI]	PPP (%)
**ARIMA terms**	AR(1)		0.11 [−0.37, 0.60]	0.11 [−0.38, 0.60]	0.06 [−0.46, 0.58]	0.09 [−0.39, 0.58]	100	0.08 [−0.43, 0.58]	100
	AR(2)		**0.57 [0.22, 0.91]**	**0.59 [0.24, 0.94]**	**0.57 [0.20, 0.93]**	**0.58 [0.23, 0.93]**	100	**0.57 [0.21, 0.93]**	100
	MA(1)		0.10 [−0.39, 0.59]	0.08 [−0.41, 0.57]	0.12 [−0.40, 0.63]	0.09 [−0.40, 0.58]	100	0.10 [−0.40, 0.61]	100
	MA(2)		−0.38 [−0.76, 0.01]	**−0.40 [−0.79, −0.01]**	**−0.37 [−0.76, −0.02]**	**−0.39 [−0.77, 0.00]**	100	−0.38 [−0.76, 0.01]	100
	MA(3)		−0.18 [−0.42, 0.05]	−0.18 [−0.43, 0.07]	−0.12 [−0.37, 0.13]	−0.16 [−0.41, 0.09]	100	−0.14 [−0.39, 0.12]	100
**Relative humidity**	3-mo.	**−0.202**				0.00 [−0.02, 0.02]	9.5	0.00 [−0.04, 0.04]	27.9
	5-mo.	**−0.239**		**−0.15 [−0.27, −0.02]**		−0.05 [−0.18, 0.09]	34.3	−0.05 [−0.22, 0.11]	50.0
**Rainfall frequency**	5-mo.	**−0.206**			**−0.11 [−0.23, 0.00]**	−0.03 [−0.14, 0.08]	29.2	−0.05 [−0.20, 0.10]	49.7
**Mean daily rainfall**	10-mo.	**0.189**			**0.12 [0.01, 0.22]**	0.04 [−0.08, 0.17]	38.8	0.08 [−0.06, 0.22]	71.9
	22-mo.	**0.187**			0.11 [−0.01, 0.22]	0.04 [−0.08, 0.16]	34.3	0.07 [−0.07, 0.21]	66.3
**MEI**	21-mo.	**0.258**				0.00 [−0.01, 0.02]	8.5	0.01 [−0.05, 0.06]	28.4
**(Intercept)**			**2.01 [1.83, 2.19]**	**2.00 [1.83, 2.17]**	**2.01 [1.84, 2.18]**	**2.00 [1.83, 2.18]**	100	**2.00 [1.83, 2.18]**	100

The values of the cross correlation function (CCF) between the pre-whitened series are presented alongside parameter estimates in the best-fitting and averaged models according to each information criterion. Significance at the 95% confidence level is indicated with **bold** text.

For the multivariate models, each covariate had the binary option of being included or not included. Since we identified six significant cross correlations, we fit 2^6^ = 64 models in total. The best-fitting model according to BIC weights accounted only for a negative association between cases and five month-lagged relative humidity. The best-fitting model by AIC and AICc included a negative association with rainfall frequency at the five-month lag and total rainfall at 10- and 21-month lags. Averaging across all models did not reveal noteworthy differences in variables' contributions to model fit, as evidenced by similarity in PPP values among covariates under each averaging scheme. Parameter estimates averaged according to AIC and AICc weights differed by less than 10^−4^ and are consequently presented together as a single averaged model. Under the BIC and AIC/AICc weighting strategies, the models with the greatest posterior probabilities accounted for relative humidity and rainfall frequency at five-month lags, and total rainfall at 10- and 21-month lags. Meteorological parameter estimates differed across models, leading to averaged 95% confidence intervals including zero in all cases. The BIC-averaged model offered more conservative estimates and narrower confidence intervals for all meteorological parameters than the AIC/AICc-averaged model. Using model weights computed from the training period only rather than monthly-updated model weights did not lead to numerical changes in parameter estimates or PPPs greater than 10^−4^. Variable selection for the best-fitting models by AIC/AICc and BIC did not change as we updated the models.

### Forecasts

**Figure 3:**
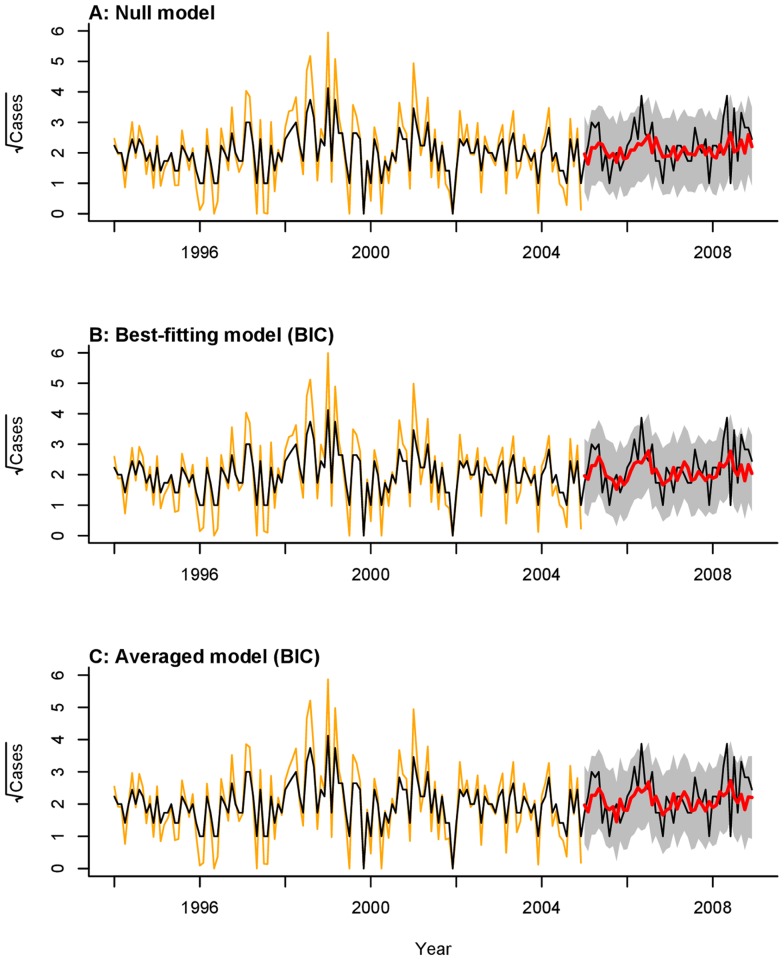
One month-ahead forecasts. (A) Null model; (B) Best-fitting model according to BIC; (C) Averaged model according to BIC. Black lines plot the square root-transformed cases; orange lines plot model fit to data during the training period; red lines plot model forecasts, with the grey area representing the 95% confidence region.

We compared out-of-fit prediction accuracy for the null model with the best-fit models and the averaged models, which accounted for meteorological covariates (**Table 2**). The best-fit and averaged models reduced the MSE relative to the null model at all prediction lengths. Improvements in MSE relative to the null model were greatest at the three-month horizon and smallest at the one-month horizon for all models considered. The best-fitting model by BIC produced one, two, and three month-ahead forecasts with 10.6%, 12.8%, and 15.7% lower MSE than the null model, respectively (**Figure 3** **, Figure S1, Figure S2**). This model provided the most accurate forecasts at all prediction lengths; two month-ahead forecasts were the most accurate in terms of minimizing MSE. The averaged model constructed according to BIC offered smaller marginal reductions in MSE than the averaged model constructed according to AIC/AICc weights for all but the one month-ahead predictive horizon. Marginal reductions in prediction MSE were poorest from the best-fitting model selected according to AIC/AICc weights.

**Table 2:** Measures of prediction error.

		Null model			Best-fit (BIC)			Averaged (BIC)			Best-fit (AIC/AICc)			Averaged (AIC/AICc)		
	Months ahead	1	2	3	1	2	3	1	2	3	1	2	3	1	2	3
**MSE**		0.531	0.524	0.578	**0.475**	**0.457**	**0.488**	**0.491**	**0.484**	**0.509**	0.509	**0.493**	**0.533**	**0.494**	**0.475**	**0.509**
**∂(MSE_0_)**					**−10.6%**	**−12.8%**	**−15.7%**	**−7.4%**	**−9.5%**	**−12.0%**	−4.1%	**−5.9%**	**−7.7%**	**−6.9%**	**−9.4%**	**−12.1%**

Mean squared error (MSE) in predictions is presented for each model at each forecast horizon (1, 2, and 3 months ahead). Percent change in MSE relative to the null model (∂MSE_0_) is presented to measure improvement in prediction accuracy. Improvements greater than 5% relative to the null model are indicated with **bold** text.

The best-fitting model by BIC offered one-, two-, and three month-ahead predictions with on average 6.0%, 7.3%, and 8.0% lower variance than the null model, respectively. These improvements in precision did not incur penalties to forecast accuracy. Observed cases exceeded the upper limits of the 95% confidence envelopes from all models in May of 2006 and May of 2008, at the peaks of epidemics during those years. One- and three month-ahead predictions from all models additionally under-estimated a secondary peak in July of 2008 (**Figure S1**, **Figure S2**). Adjusting models to include a seasonal autoregressive for the twelve-month period term did not improve forecasting of the May epidemic peaks, which became a regular feature in the data only from 2005 onward. Residuals from models incorporating covariates did not show significant temporal dependence via the Ljung-Box and test or in their autocorrelation and partial autocorrelation functions.

## Discussion

In this study we found that accounting for meteorological and climatic factors improved accuracy and precision of CL forecasts in a region of endemic *L. (V.) braziliensis* transmission in Northeast Brazil. Notably, dry conditions with respect to relative humidity and precipitation were significantly associated with CL case notifications three to five months later. Our results are consistent with the view that CL is sensitive to meteorological and climatic forcing [19], [22], [30]–.

Differences in out-of-fit predictive accuracy among models likely indicate where models may be overfit to within-sample data. The model with the best predictive accuracy at all horizons was selected by BIC and accounted only for five month-lagged relative humidity as a meteorological covariate. AIC and AICc have a lower penalty than BIC for potential overfitting [40], [41], and in the present analysis selected a model with more covariates, including covariates operating at longer (10- and 21-month) lags. Temporally remote effects of this nature may be difficult to identify and use for prediction due to heterogeneity in CL incubation periods [47], in the time individuals take to seek medical attention, and in ecological pathways connecting weather to disease risk. These factors contribute to uncertainty when forecasting with case notification data [48].

Although our analysis was not suited for identification of causal effects, numerous biological mechanisms may support associations between weather and CL epidemics. Inverse correlations between precipitation and humidity variables at lags between three and five months in particular demonstrate excess cases closely follow dry periods. Ecological sampling studies have indicated population densities of *Lutzomyia* sandflies in CL-endemic areas of Brazil and South America to be inversely correlated with relative humidity and rainfall in recent months [49]–[51]. While present year-round, dominant vectors for CL in the study region (including *Lu. whitmani* and *Lu. migonei*) are particularly abundant during the warm, dry season [23], [50]. The near-term moisture effects we identify may thus result from environmental conditions conducive to vector survival, reproduction, or feeding behavior. Mechanisms connecting weather and CL risk at longer lags are more likely mediated by the ecology of vertebrate *L. (V.) braziliensis* host species than by sandflies, whose life cycles span only one to several months. For instance, the positive cross-correlations with rainfall and MEI at 21- and 22-month lags most probably relate to longer-term effects of moisture surpluses on biological productivity necessary to sustain large populations of mammalian reservoirs [25], [52].

Our analyses have several limitations. Having fit models to a 10% subset of total reported cases treated at the health post, our estimates are sensitive to small month-to-month variations that may be less pronounced in a dataset providing complete case records. While forecasts succeeded in predicting overall epidemic patterns exhibited from 2005 to 2008, a notable weakness was the poor prediction of the size and duration of the 2008 epidemic. The low number of cases appearing in June of that year, mid-way through the epidemic, may be an artifact of the reduced dataset and likely contributed to this shortcoming. The passive surveillance system at the Corte de Pedra health post additionally provided an incomplete sample of total CL cases within the area, and may in particular under-represent persons unlikely to seek care. For instance, other analyses of the data presented here showed that many agricultural workers delay pursuit of therapy until onset of complications including mucosal or disseminated infection [7]. If long or heterogeneous gaps separate timing of infection, disease onset, and care-seeking, case notification data may not indicate sharp peaks in CL following important weather events. Such bias obscures potentially meaningful meteorological associations with CL risk.

While the Corte de Pedra health post remained the primary center for CL diagnosis and treatment throughout the study period [7], [33], [34], there were almost certainly long-term changes among the population at risk with respect to size, demographics, access to health services, and potential environmental exposures. Our analysis could not address these factors as potential controls or effect modifiers because the health post serves small rural communities not tabulated by the Brazilian census. Ongoing deforestation in the region, including conversion of cacao plantations to cattle ranches, likely caused temporal variation in habitat suitability for vectors and hosts and thus mediated disease risk. In addition, Northeast Brazil experienced secular rural-to-urban migration during the study period, likely offsetting natural increase within the population. Individual risk factors likewise predict variation within the population with respect to opportunities for exposure to *L. (V.) braziliensis* in domestic, peridomestic, and sylvatic environments [7]. Consequently, individual factors not considered here may mediate temporal patterns and weather sensitivity of CL risk among patients [23], [25], [53]. In view of these limitations to the present study, implications of population- and individual-level factors for CL transmission require future research attention to inform interventions reducing disease risk in northeastern Brazil.

Although the identified associations aided forecasting, inferential gains are limited by poor understanding of CL eco-epidemiology, including the fact that the local animal reservoir for *L. (V.) braziliensis* is unknown. Ecological sampling studies in the state of Pernambuco, near the study region, suggest several species of mice and rats may contribute to transmission [18], however the parasite is known to infect other rodents as well as dogs, cats, and equines [54]. Potential pathways by which weather affects ecological dynamics in American CL have been discussed extensively in previous work [55], [56], and are likely geographically heterogeneous. Meteorological and climatic sensitivity of *Leishmania spp.* transmission cycles can be anticipated to vary spatially according to species compositions, contact rates and competence among local vectors and hosts, and ecological sensitivity to weather and other environmental stressors; additionally, individually- and regionally-varying social factors influence human exposure to primarily-enzootic transmission cycles, and vulnerability to weather-related health risks [22], [57]. It is known, for instance, that seasonal dynamics of *L. (V.) braziliensis* and its vectors differ across Brazil, where predominantly sylvatic, peridomestic, or domestic transmission pathways in endemic foci reflect divergent underpinnings of CL eco-epidemiology [26], [29], [58]. For this reason, developing similar model-based early warning systems at fine geographic resolutions remains an important objective for other endemic settings within Brazil and Latin America.

As CL continues to expand in parts of Brazil, developing capacity to forecast epidemics will facilitate public health responses. Using model-based predictions to anticipate disease risk and expanding clinical capacity to address excess CL cases may constitute an important operational strategy for alleviating burden of disease. For example, understanding the timing of epidemics will enable implementation of enhanced case detection in advance of and during high-risk periods, limiting lesion size at the time patients are identified and reducing patients' risk for treatment failure and metastatic complications. This can be accomplished in part by ensuring adequate clinical and laboratory personnel and diagnostic reagents or microscopy resources are available to identify CL patients during high-demand periods. Furthermore, since procurement and delivery of first-line pentavalent antimonial agents to endemic regions requires significant lead-time, acquiring and distributing these drugs preemptively in response to model-based predictions may ensure that treatment centers are adequately stocked for epidemics. This is also critical with respect to maintaining supplies of difficult-to-procure alternative therapies such as liposomal amphotericin B [59], which may need to be considered as *L. (V.) braziliensis* strains resistant to conventional treatments continue to emerge [7], [12]–[14]. Spatial and population-based criteria merit consideration in service delivery so that clinical resources and surveillance attention can be targeted focally towards vicinities or persons known to be at high risk for infection [60], [61]; in the study area, this population primarily includes young men who work or live in agricultural settings [7], [8], [14].

One key question with respect to the application of model-based forecasting to improve responses to CL is the definition of epidemic thresholds. The limited capacities of local and national leishmaniasis control programs in resource-poor settings contribute to difficulty identifying alert and response priorities for early warning systems [62], particularly with respect to defining meaningful epidemic thresholds. Choice of such thresholds may be arbitrary in practice [63]. WHO standards for initiating alerts following months when incidence has been twice its monthly average are likely sub-optimal for settings with highly variable incidence rates, such as Northeast Brazil [64], [65], where doubling relative to previous monthly averages may not be an adequate basis for identifying an epidemic and anticipating whether it will continue. More meaningful intervention criteria in endemic regions may be based on model-predicted probabilities for incidence to exceed a level at which clinical resources are likely to be strained; probabilistic alert systems of this nature are increasingly recognized for their compatibility with model-based epidemic projections, and interpretable implications for policy responses [66]. Operational research is needed to assess how clinical capacity and resilience to epidemics vary across endemic settings, as a basis for setting alert thresholds informed by risk for shortcomings in service delivery. Notwithstanding these limitations to operationalizing early warning systems in Brazil, our outcomes suggest that incoming weather data improves CL forecasts at a sufficient predictive horizon to facilitate intervention planning. Best practices for integrating predictive models into planning for responses to CL epidemics merit research attention and consideration from public health authorities in CL-endemic areas [15], [19], [67], [68].

## Supporting Information

Figure S1 **Two month-ahead forecasts.** (A) Null model; (B) Best-fitting model according to BIC; (C) Averaged model according to AIC/AICc. Black lines plot the square root-transformed cases; orange lines plot model fit to data during the training period; red lines plot model forecasts, with the grey area representing the 95% confidence region.
(TIFF)

Figure S2 **Three month-ahead forecasts.** (A) Null model; (B) Best-fitting model according to BIC; (C) Averaged model according to AIC/AICc. Black lines plot the square root-transformed cases; orange lines plot model fit to data during the training period; red lines plot model forecasts, with the grey area representing the 95% confidence region.
(TIFF)

Table S1 **Municipality locations.**
(DOCX)

Text S1 **Weather interpolation.**
(PDF)

Data file S1 **Case series.** The file contains the data used for the study (CL cases treated at the Corte de Pedra health post, aggregated by month).
(CSV)
