# Supplementary figures and images for: Forecasting Temporal Dynamics of Cutaneous Leishmaniasis in Northeast Brazil
Source: PLoS Negl Trop Dis. 2014 Oct 30;8(10):e3283. doi: 10.1371/journal.pntd.0003283 (PMC4214672; doi:10.1371/journal.pntd.0003283)

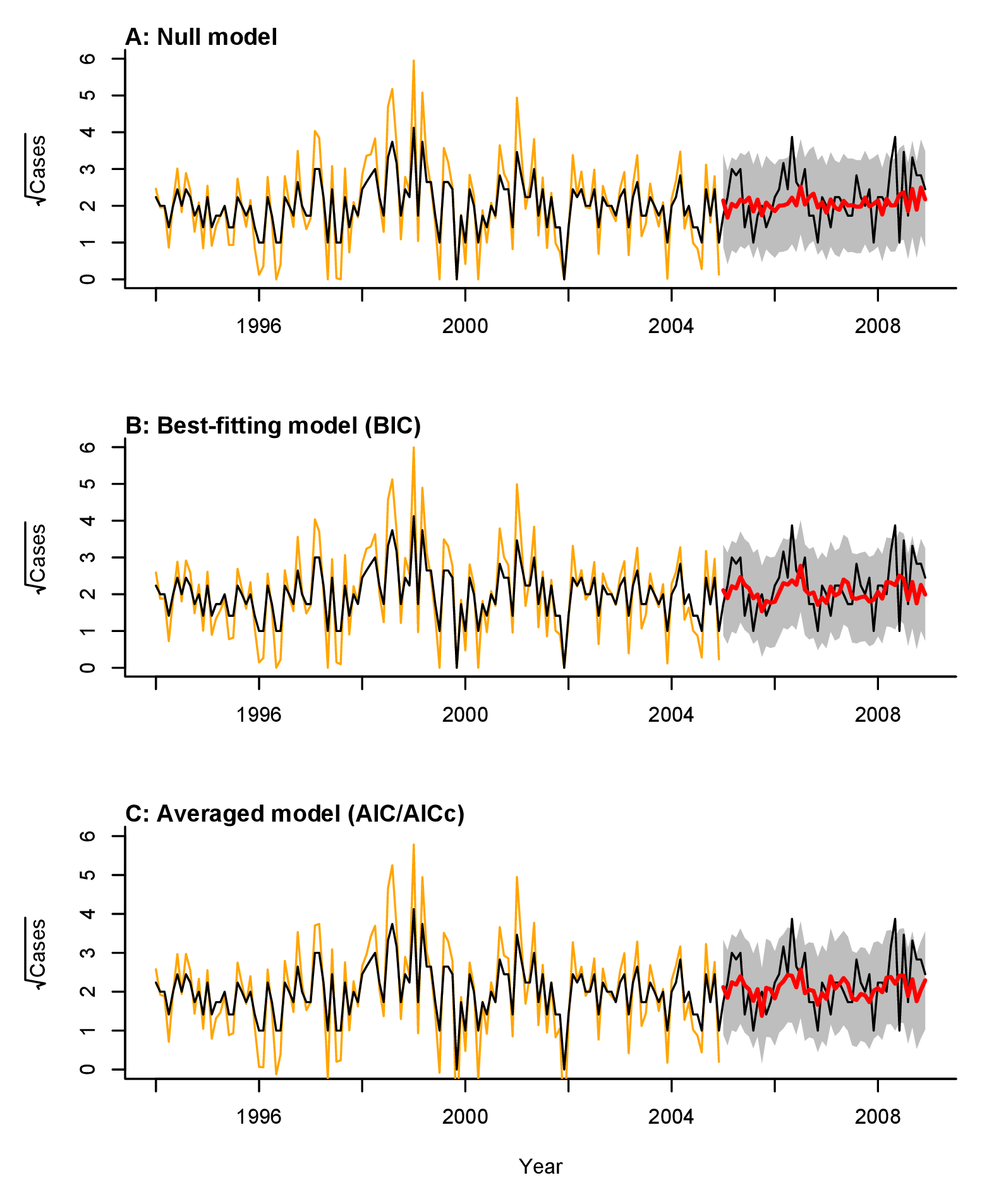

Supplement: Figure S1 — Two month-ahead forecasts. (A) Null model; (B) Best-fitting model according to BIC; (C) Averaged model according to AIC/AICc. Black lines plot the square root-transformed cases; orange lines plot model fit to data during the training period; red lines plot model forecasts, with the grey area representing the 95% confidence region. (TIFF) [file pntd.0003283.s001.tiff]

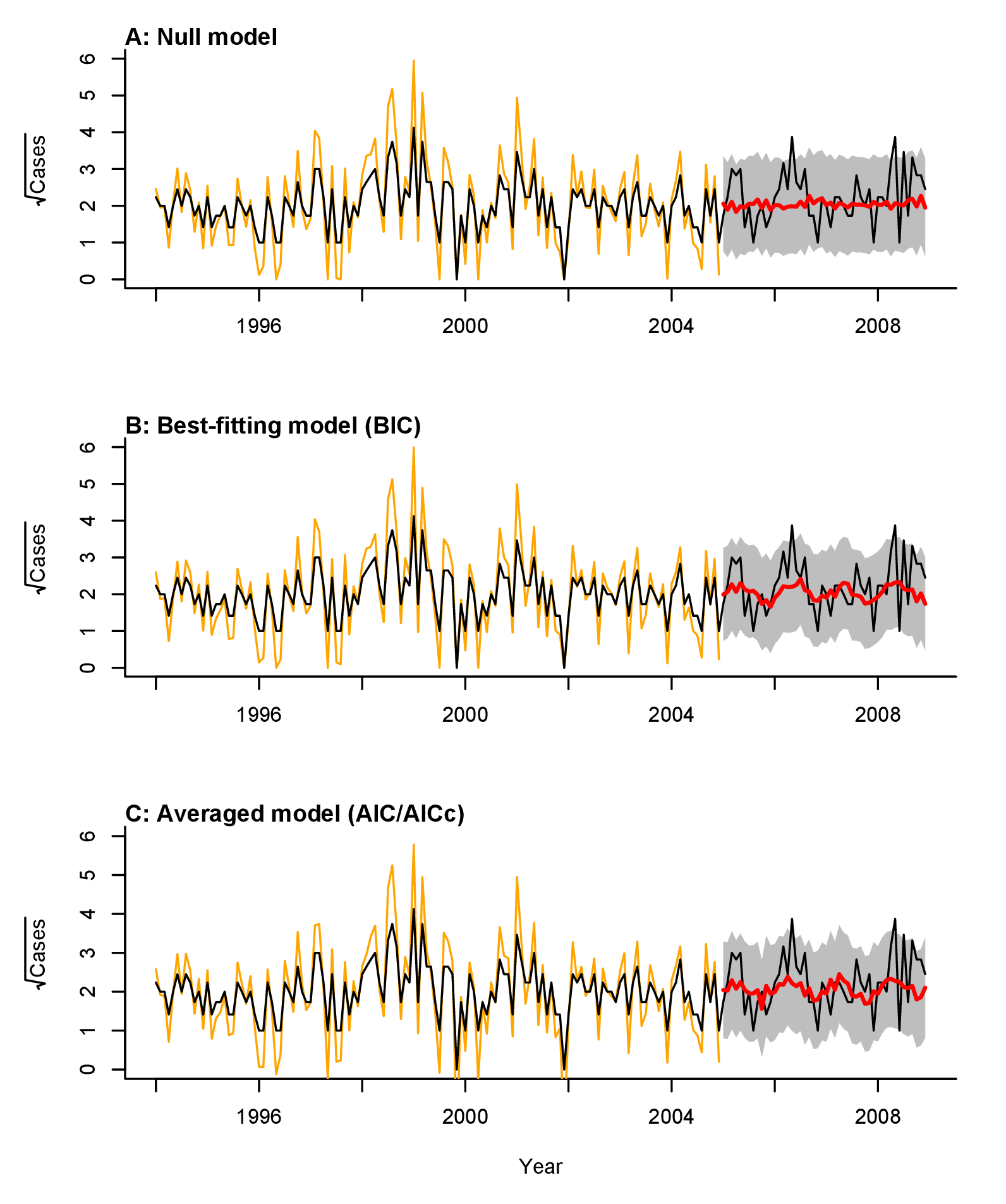

Supplement: Figure S2 — Three month-ahead forecasts. (A) Null model; (B) Best-fitting model according to BIC; (C) Averaged model according to AIC/AICc. Black lines plot the square root-transformed cases; orange lines plot model fit to data during the training period; red lines plot model forecasts, with the grey area representing the 95% confidence region. (TIFF) [file pntd.0003283.s002.tiff]
