# Supplementary material for: Forecasting Temporal Dynamics of Cutaneous Leishmaniasis in Northeast Brazil
Source: PLoS Negl Trop Dis. 2014 Oct 30;8(10):e3283. doi: 10.1371/journal.pntd.0003283 (PMC4214672; doi:10.1371/journal.pntd.0003283)
Supplement: Table S1 — Municipality locations. (DOCX) [file pntd.0003283.s003.docx]

**Table S1. Municipality Haversine distances to weather stations (km)**

|  | Station: | 83190 | 83222 | 83229 | 83244 | 83249 | 83292 | 83295 | 83344 | 83096 | 83195 | 83398 |
| --- | --- | --- | --- | --- | --- | --- | --- | --- | --- | --- | --- | --- |
| Municipality | *Elev. (m)* | *(360)* | *(225)* | *(51)* | *(250)* | *(131)* | *(531)* | *(755)* | *(839)* | *(5)* | *(208)* | *(4)* |
| Presidente Tancredo Neves | *(253)* | 208 | 95 | 109 | 149 | 160 | 207 | 58 | 319 | 382 | 313 | 252 |
| Wenceslau Guimarães | *(189)* | 235 | 122 | 128 | 163 | 186 | 197 | 62 | 293 | 406 | 338 | 227 |
| Teolândia | *(235)* | 225 | 111 | 122 | 155 | 177 | 198 | 56 | 303 | 398 | 329 | 238 |
| Mutuipe | *(423)* | 184 | 75 | 109 | 124 | 145 | 206 | 52 | 339 | 369 | 296 | 280 |
| Ibirapitanga | *(113)* | 84 | 168 | 157 | 211 | 226 | 210 | 107 | 257 | 440 | 378 | 175 |
| Itamarí | *(282)* | 251 | 140 | 153 | 159 | 207 | 174 | 54 | 273 | 428 | 359 | 225 |
| Gandú | *(229)* | 242 | 128 | 133 | 168 | 192 | 196 | 66 | 287 | 412 | 345 | 221 |
| Piraí do Norte | *(172)* | 38 | 123 | 123 | 175 | 185 | 206 | 75 | 294 | 403 | 337 | 219 |
| Valença | *(39)* | 193 | 78 | 72 | 174 | 133 | 246 | 95 | 347 | 350 | 285 | 256 |
| Taperoá | *(31)* | 212 | 97 | 86 | 181 | 151 | 240 | 94 | 330 | 367 | 303 | 237 |
| Nilo Peçanha | *(52)* | 220 | 104 | 91 | 186 | 158 | 238 | 95 | 324 | 373 | 310 | 230 |
| Ituberá | *(62)* | 234 | 119 | 105 | 191 | 173 | 232 | 96 | 309 | 387 | 325 | 216 |

Stations identified by World Meteorological Organization number
