# Supplementary material for: Forecasting Temporal Dynamics of Cutaneous Leishmaniasis in Northeast Brazil
Source: PLoS Negl Trop Dis. 2014 Oct 30;8(10):e3283. doi: 10.1371/journal.pntd.0003283 (PMC4214672; doi:10.1371/journal.pntd.0003283)
Supplement: Text S1 — Weather interpolation. (PDF) [file pntd.0003283.s004.pdf]

Supplemental materials

S2. Weather interpolation

We computed mean monthly meteorological conditions within each region for the extent of the case time series via a spatially-weighted, elevation-based deterministic interpolation procedure (see Thornton 1997). We identified that observation-days were missing completely at random from stations reporting to INMET via Little's test, and calculated a simple mean value for each month across daily reported noontime temperature, relative humidity, rainfall occurrence, and total rainfall at each station. For each variable we additionally generated vectors indicating data completeness rate, defined as the monthly mean of an indicator variable for daily reporting. Values ranged from 90–100% for observation-months across all variables, however were zero for 7% of relative humidity observation-months.

We interpolated monthly mean conditions at the main municipalities within each region (listed in Supplemental table S1). We defined a spatial weight w for each station and variable relative to each municipality according to the station Haversine distance r via a Gaussian kernel function truncated at $R = 500$km, and downweighted by reporting completeness:

$$w_i = \left[\exp\Big(-\alpha\big(r_i/R\big)^2\Big) - \exp\big(-\alpha\big)\right] \times \mathbb{E}\Big(\mathbb{I}\big\{X_{(i,t)} \text{ observed}\big\}\Big) \tag{1}$$

We arbitrarily defined $\alpha = 1$ and identified that interpolated values were insensitive to changes in α within one order of magnitude. We fit a saturated linear model to serve as a basis for interpolation by regressing differences in observations between stations $X_i - X_j$ as a function of their elevation difference $Z_i - Z_j$, weighting observations by the products $w_i w_j$ for all $i \neq j$ station-month pairings. We allowed for main effects and interaction terms by calendar month, thus fitting parameters to define:

$$\mathbb{E}\big(X_i - X_j\big) = \frac{\sum_{i\neq j} w_i w_j \hat{\Delta}_{(i,j)}}{\sum_{i\neq j} w_i w_j}$$

$$\text{where } \hat{\Delta}_{(i,j)} = \beta_0 + \beta_1\big(Z_i - Z_j\big) + \sum_{m=1}^{11}\left[\mathbb{I}\big\{\text{Month} = m\big\}\Big(\beta_{(2,m)} + \beta_{(3,m)}\big(Z_i - Z_j\big)\Big)\right]$$

We used the fitted parameter values to generate point estimates for each month at each municipality based on observed conditions at each station. We pooled point estimates for each municipality as a spatially-weighted average across the estimate generated from each station, with weights estimated by the Gaussian kernel as above.
